# 2-(4-Bromo­phen­yl)-*N*-(3,4-di­fluoro­phen­yl)acetamide

**DOI:** 10.1107/S1600536813012865

**Published:** 2013-05-18

**Authors:** A.S. Praveen, H. S. Yathirajan, Jerry P. Jasinski, Amanda C. Keeley, B. Narayana, B. K. Sarojini

**Affiliations:** a Department of Studies in Chemistry, University of Mysore, Manasagangotri, Mysore 570 006, India; b Department of Chemistry, Keene State College, 229 Main Street, Keene, NH 03435-2001, USA; c Department of Studies in Chemistry, Mangalore University, Mangalagangotri 574 199, India; d Department of Chemistry, P.A.College of Engineering, Nadupadavu, Mangalore 574 153, India

## Abstract

In the title compound, C_14_H_10_BrF_2_NO, the dihedral angle between the mean planes of the 4-bromo­phenyl and 3,4-di­fluoro­phenyl rings is 66.4 (1)°. These two planes are twisted by 40.0 (5) and 86.3 (2)°, respectively, from that of the acetamide group. In the crystal, N—H⋯O hydrogen bonds and weak C—H⋯O and C—H⋯F inter­actions form infinite chains along [100].

## Related literature

For the structural similarity of *N*-substituted 2-aryl­acetamides to the lateral chain of natural benzyl­penicillin, see: Mijin & Marinkovic (2006 ▶); Mijin *et al.* (2008 ▶). For the coordination abilities of amides, see: Wu *et al.* (2008 ▶, 2010 ▶). For related structures, see: Praveen *et al.* (2011*a* ▶,*b* ▶,*c* ▶, 2012 ▶). For standard bond lengths, see: Allen *et al.* (1987 ▶).
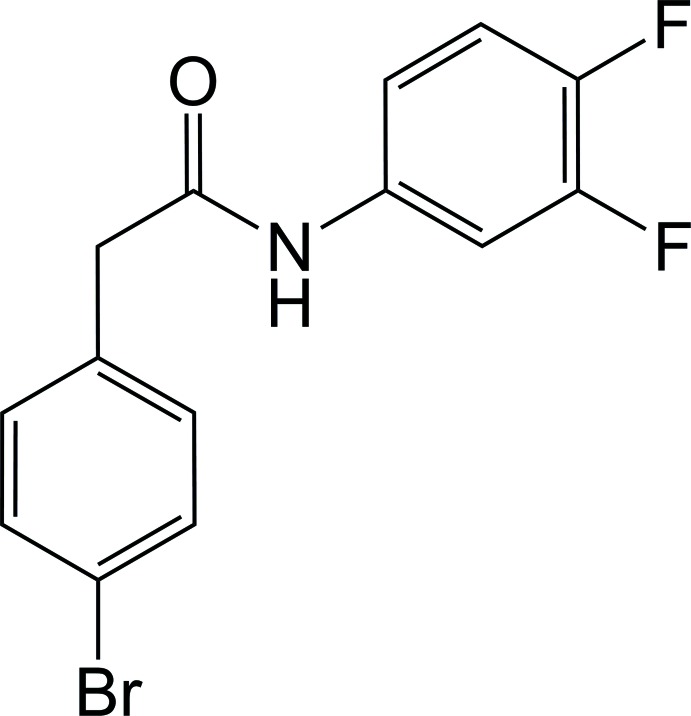


## Experimental

### 

#### Crystal data

C_14_H_10_BrF_2_NO
*M* *_r_* = 326.14
Orthorhombic, 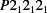

*a* = 4.9136 (3) Å
*b* = 6.0218 (4) Å
*c* = 42.514 (2) Å
*V* = 1257.96 (13) Å^3^
*Z* = 4
Cu *K*α radiation
μ = 4.62 mm^−1^
*T* = 173 K
0.48 × 0.32 × 0.16 mm

#### Data collection

Agilent Xcalibur (Eos, Gemini) diffractometer
Absorption correction: multi-scan (*CrysAlis PRO* and *CrysAlis RED*; Agilent, 2012 ▶) *T*_min_ = 0.257, *T*_max_ = 1.000
6592 measured reflections
2402 independent reflections
2388 reflections with *I* > 2σ(*I*)
*R*_int_ = 0.036

#### Refinement

*R*[*F* ^2^ > 2σ(*F* ^2^)] = 0.041
*wR*(*F* ^2^) = 0.106
*S* = 1.15
2402 reflections
173 parameters
H-atom parameters constrained
Δρ_max_ = 0.87 e Å^−3^
Δρ_min_ = −0.59 e Å^−3^
Absolute structure: Flack *x* determined using 915 quotients [(*I* ^+^)−(*I* ^−^)]/[(*I* ^+^)+(*I* ^−^)] (Parsons & Flack, 2004 ▶)
Flack parameter: −0.01 (2)

### 

Data collection: *CrysAlis PRO* (Agilent, 2012 ▶); cell refinement: *CrysAlis PRO*; data reduction: *CrysAlis RED* (Agilent, 2012 ▶); program(s) used to solve structure: *SHELXS97* (Sheldrick, 2008 ▶); program(s) used to refine structure: *SHELXL2012* (Sheldrick, 2008 ▶); molecular graphics: *OLEX2* (Dolomanov *et al.*, 2009 ▶); software used to prepare material for publication: *OLEX2*.

## Supplementary Material

Crystal structure: contains datablock(s) global, I. DOI: 10.1107/S1600536813012865/hg5313sup1.cif

Structure factors: contains datablock(s) I. DOI: 10.1107/S1600536813012865/hg5313Isup2.hkl

Supplementary material file. DOI: 10.1107/S1600536813012865/hg5313Isup3.cml

Additional supplementary materials:  crystallographic information; 3D view; checkCIF report

## Figures and Tables

**Table 1:** Hydrogen-bond geometry (Å, °)

*D*—H⋯*A*	*D*—H	H⋯*A*	*D*⋯*A*	*D*—H⋯*A*
N1—H1⋯O1^i^	0.88	1.97	2.851 (7)	175
C7—H7⋯O1^ii^	0.95	2.63	3.309 (8)	129
C13—H13⋯F1^iii^	0.95	2.50	3.426 (9)	164

Symmetry codes: (i) 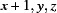; (ii) 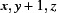; (iii) 
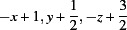
.

**Fig. 1.**
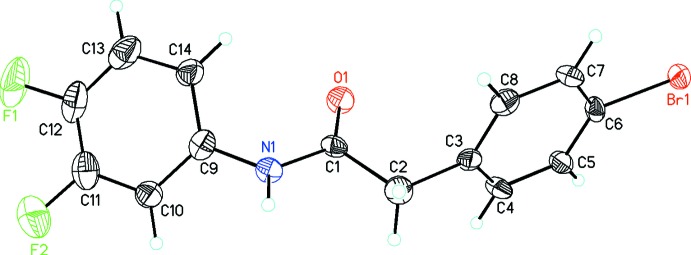
Molecular structure of the title compound showing the atom labeling scheme and 30% probability displacement ellipsoids.

**Fig. 2.**
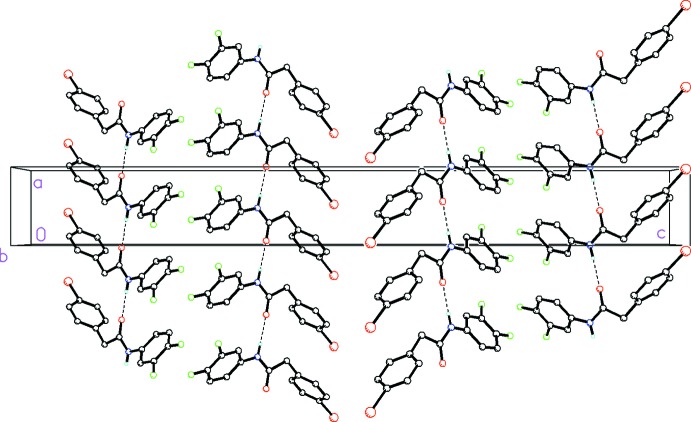
Packing diagram of the title compound viewed along the b axis. Dashed lines indicate N—H···O hydrogen bonds forming infinite chains along (100). H atoms not involved in hydrogen bonding have been deleted for clarity.

**Fig. 3.**
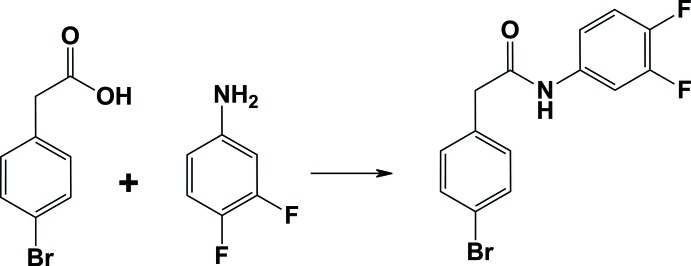
Synthesis of (I).

## supplementary crystallographic information

### Comment

N-Substituted 2-arylacetamides are very interesting compounds because of their structural similarity to the lateral chain of natural benzylpenicillin (Mijin *et al.*, 2006, 2008). Amides are also used as ligands due to their excellent coordination abilities (Wu *et al.*, 2008, 2010). Crystal structures of some acetamide derivatives viz., N-(3-chloro-4-fluorophenyl)-2-(naphthalen-1-yl)acetamide (Praveen *et al.*, 2011*a*), N-(4-chloro-1,3-benzothiazol-2-yl)-2- (3-methylphenyl)acetamide monohydrate (Praveen *et al.*, 2011*b*), N-(3-chloro-4-fluorophenyl)-2,2-diphenylacetamide (Praveen *et al.*, 2011*c*) and N-(4,6-dimethoxypyrimidin-2-yl)-2-(3-methylphenyl)acetamide (Praveen *et al.*, 2012) have been reported. In view of the importance of amides, we report here in the crystal structure of the title compound, C_14_H_10_BrF_2_NO, (I).

In (I) the dihedral angle between the mean planes of the 4-bromophenyl and 3,4-difluorophenyl rings is 66.4 (1)° (Fig. 1). These two planes are twisted by 40.0 (5)° and 86.3 (2)°, respectively, from that of the acetamide group. Bond lengths are in normal ranges (Allen *et al.*, 1987). In the crystal, N—H···O hydrogen bonds and weak C—H···O and C—H···F intermolecular interactions are observed forming a infinite chains along (100) and contribute to packing stability (Fig. 2).

### Experimental

4-bromophenylacetic acid (0.213 g, 1 mmol), 3,4-difluoro aniline (0.129 g, 1 mmol) and 1-ethyl-3-(3-dimethylaminopropyl)carbodiimide hydrochloride (1.0 g, 0.01 mol) were dissolved in dichloromethane (20 mL) (Fig. 3). The mixture was stirred in presence of triethylamine at 273 K for about 3 h. The contents were poured into 100 ml of ice-cold aqueous hydrochloric acid with stirring, which was extracted thrice with dichloromethane. The organic layer was washed with saturated NaHCO_3_ solution and brine solution, dried and concentrated under reduced pressure to give the title compound (I). Single crystals were grown from methylene chloride by the slow evaporation method (m.p.: 423–425 K).

### Refinement

All of the H atoms were placed in their calculated positions and then refined using the riding model with Atom—H lengths of 0.95Å (CH), 0.99Å (CH_2_) or 0.88° (NH). Isotropic displacement parameters for these atoms were set to 1.2 (CH, CH_2_, NH) times *U*_eq_ of the parent atom.

### Crystal data

**Table:**

C_14_H_10_BrF_2_NO	*D*_x_ = 1.722 Mg m^−^^3^
*M**_r_* = 326.14	Cu *K*α radiation, λ = 1.54184 Å
Orthorhombic, *P*2_1_2_1_2_1_	Cell parameters from 3482 reflections
*a* = 4.9136 (3) Å	θ = 3.1–71.3°
*b* = 6.0218 (4) Å	µ = 4.62 mm^−^^1^
*c* = 42.514 (2) Å	*T* = 173 K
*V* = 1257.96 (13) Å^3^	Block, colorless
*Z* = 4	0.48 × 0.32 × 0.16 mm
*F*(000) = 648	

### Data collection

**Table:**

Agilent Xcalibur (Eos, Gemini) diffractometer	2402 independent reflections
Radiation source: Enhance (Cu) X-ray Source	2388 reflections with *I* > 2σ(*I*)
Detector resolution: 16.1500 pixels mm^-1^	*R*_int_ = 0.036
ω scans	θ_max_ = 71.4°, θ_min_ = 4.2°
Absorption correction: multi-scan (*CrysAlis PRO* and *CrysAlis RED*; Agilent, 2012)	*h* = −5→4
*T*_min_ = 0.257, *T*_max_ = 1.000	*k* = −7→7
6592 measured reflections	*l* = −51→52

### Refinement

**Table:**

Refinement on *F*^2^	H-atom parameters constrained
Least-squares matrix: full	*w* = 1/[σ^2^(*F*_o_^2^) + (0.0518*P*)^2^ + 2.6477*P*] where *P* = (*F*_o_^2^ + 2*F*_c_^2^)/3
*R*[*F*^2^ > 2σ(*F*^2^)] = 0.041	(Δ/σ)_max_ = 0.001
*wR*(*F*^2^) = 0.106	Δρ_max_ = 0.87 e Å^−^^3^
*S* = 1.15	Δρ_min_ = −0.59 e Å^−^^3^
2402 reflections	Extinction correction: *SHELXL2012* (Sheldrick, 2008), Fc^*^=kFc[1+0.001xFc^2^λ^3^/sin(2θ)]^-1/4^
173 parameters	Extinction coefficient: 0.0034 (5)
0 restraints	Absolute structure: Flack *x* determined using 915 quotients [(*I*^+^)-(*I*^-^)]/[(*I*^+^)+(*I*^-^)] (Parsons & Flack, 2004)
Hydrogen site location: inferred from neighbouring sites	Flack parameter: −0.01 (2)

### Special details

**Table:**

Geometry. All esds (except the esd in the dihedral angle between two l.s. planes) are estimated using the full covariance matrix. The cell esds are taken into account individually in the estimation of esds in distances, angles and torsion angles; correlations between esds in cell parameters are only used when they are defined by crystal symmetry. An approximate (isotropic) treatment of cell esds is used for estimating esds involving l.s. planes.

### Fractional atomic coordinates and isotropic or equivalent isotropic displacement parameters (Å^2^)

**Table:**

	*x*	*y*	*z*	*U*_iso_*/*U*_eq_	
Br1	0.05444 (13)	1.14849 (10)	0.52705 (2)	0.0255 (2)	
F1	0.8291 (12)	−0.2536 (11)	0.73852 (13)	0.0682 (16)	
F2	1.2315 (12)	−0.3056 (8)	0.69684 (13)	0.0577 (14)	
O1	0.5535 (9)	0.4454 (8)	0.63179 (10)	0.0308 (9)	
N1	0.9924 (9)	0.3661 (9)	0.64466 (11)	0.0243 (11)	
H1	1.1630	0.3988	0.6404	0.029*	
C1	0.7964 (12)	0.4695 (10)	0.62744 (13)	0.0203 (12)	
C2	0.9134 (13)	0.6097 (10)	0.60058 (15)	0.0283 (13)	
H2A	1.0489	0.7148	0.6093	0.034*	
H2B	1.0088	0.5109	0.5856	0.034*	
C3	0.6979 (12)	0.7387 (11)	0.58317 (14)	0.0222 (12)	
C4	0.5905 (13)	0.6561 (11)	0.55519 (14)	0.0274 (12)	
H4	0.6491	0.5161	0.5474	0.033*	
C5	0.3960 (12)	0.7791 (10)	0.53850 (14)	0.0231 (13)	
H5	0.3216	0.7238	0.5194	0.028*	
C6	0.3155 (12)	0.9796 (10)	0.55018 (13)	0.0206 (11)	
C7	0.4148 (13)	1.0645 (10)	0.57804 (14)	0.0235 (12)	
H7	0.3531	1.2035	0.5858	0.028*	
C8	0.6072 (12)	0.9412 (11)	0.59439 (13)	0.0255 (13)	
H8	0.6779	0.9970	0.6136	0.031*	
C9	0.9424 (13)	0.2099 (10)	0.66883 (13)	0.0247 (12)	
C10	1.1086 (13)	0.0238 (12)	0.67050 (15)	0.0300 (14)	
H10	1.2496	0.0018	0.6555	0.036*	
C11	1.0656 (16)	−0.1289 (11)	0.69426 (16)	0.0365 (15)	
C12	0.8674 (15)	−0.0986 (14)	0.71582 (17)	0.0427 (19)	
C13	0.7024 (15)	0.0831 (15)	0.71475 (16)	0.0415 (19)	
H13	0.5641	0.1022	0.7301	0.050*	
C14	0.7366 (14)	0.2408 (13)	0.69109 (15)	0.0305 (14)	
H14	0.6217	0.3675	0.6901	0.037*	

### Atomic displacement parameters (Å^2^)

**Table:**

	*U*^11^	*U*^22^	*U*^33^	*U*^12^	*U*^13^	*U*^23^
Br1	0.0229 (3)	0.0270 (3)	0.0266 (3)	0.0051 (2)	0.0000 (2)	0.0054 (2)
F1	0.062 (3)	0.089 (4)	0.054 (3)	−0.020 (3)	−0.005 (3)	0.045 (3)
F2	0.063 (3)	0.045 (3)	0.065 (3)	0.010 (2)	−0.009 (3)	0.009 (2)
O1	0.015 (2)	0.045 (2)	0.032 (2)	0.003 (2)	0.0007 (18)	0.0076 (19)
N1	0.007 (2)	0.037 (3)	0.029 (2)	−0.001 (2)	0.0003 (16)	0.006 (2)
C1	0.011 (3)	0.025 (3)	0.025 (3)	0.001 (2)	0.000 (2)	−0.002 (2)
C2	0.016 (3)	0.031 (3)	0.038 (3)	−0.005 (3)	0.005 (2)	0.009 (2)
C3	0.012 (3)	0.030 (3)	0.024 (3)	0.002 (2)	0.004 (2)	0.006 (2)
C4	0.026 (3)	0.024 (3)	0.032 (3)	0.009 (3)	0.005 (2)	−0.002 (2)
C5	0.023 (3)	0.022 (3)	0.024 (3)	0.004 (2)	0.000 (2)	−0.002 (2)
C6	0.013 (3)	0.024 (3)	0.024 (3)	0.002 (2)	−0.003 (2)	0.007 (2)
C7	0.021 (3)	0.023 (3)	0.027 (3)	0.006 (3)	0.002 (2)	−0.005 (2)
C8	0.024 (3)	0.030 (3)	0.022 (3)	−0.003 (3)	0.001 (2)	−0.002 (2)
C9	0.017 (3)	0.033 (3)	0.024 (3)	−0.010 (3)	−0.006 (2)	0.003 (2)
C10	0.022 (3)	0.040 (4)	0.027 (3)	0.003 (3)	0.000 (2)	0.003 (3)
C11	0.038 (4)	0.031 (3)	0.041 (3)	−0.006 (4)	−0.013 (3)	0.006 (3)
C12	0.036 (4)	0.057 (5)	0.035 (3)	−0.016 (3)	−0.012 (3)	0.020 (3)
C13	0.025 (4)	0.069 (6)	0.029 (3)	−0.009 (4)	0.001 (3)	0.007 (3)
C14	0.024 (3)	0.042 (4)	0.025 (3)	0.002 (3)	0.001 (2)	0.003 (3)

### Geometric parameters (Å, º)

**Table:**

Br1—C6	1.909 (6)	C5—C6	1.364 (9)
F1—C12	1.356 (8)	C5—H5	0.9500
F2—C11	1.345 (9)	C6—C7	1.379 (8)
O1—C1	1.217 (8)	C7—C8	1.389 (9)
N1—C1	1.360 (7)	C7—H7	0.9500
N1—C9	1.414 (8)	C8—H8	0.9500
N1—H1	0.8800	C9—C10	1.388 (10)
C1—C2	1.532 (8)	C9—C14	1.398 (9)
C2—C3	1.508 (8)	C10—C11	1.382 (9)
C2—H2A	0.9900	C10—H10	0.9500
C2—H2B	0.9900	C11—C12	1.350 (11)
C3—C8	1.383 (9)	C12—C13	1.362 (12)
C3—C4	1.393 (9)	C13—C14	1.394 (10)
C4—C5	1.402 (8)	C13—H13	0.9500
C4—H4	0.9500	C14—H14	0.9500
			
C1—N1—C9	124.9 (5)	C6—C7—C8	118.2 (5)
C1—N1—H1	117.5	C6—C7—H7	120.9
C9—N1—H1	117.5	C8—C7—H7	120.9
O1—C1—N1	123.9 (6)	C3—C8—C7	121.2 (6)
O1—C1—C2	123.2 (6)	C3—C8—H8	119.4
N1—C1—C2	112.8 (5)	C7—C8—H8	119.4
C3—C2—C1	112.7 (5)	C10—C9—C14	119.9 (6)
C3—C2—H2A	109.0	C10—C9—N1	118.2 (6)
C1—C2—H2A	109.0	C14—C9—N1	122.0 (6)
C3—C2—H2B	109.0	C11—C10—C9	119.0 (6)
C1—C2—H2B	109.0	C11—C10—H10	120.5
H2A—C2—H2B	107.8	C9—C10—H10	120.5
C8—C3—C4	119.2 (6)	F2—C11—C12	119.3 (6)
C8—C3—C2	120.7 (6)	F2—C11—C10	119.6 (7)
C4—C3—C2	120.1 (6)	C12—C11—C10	121.1 (7)
C3—C4—C5	120.1 (6)	C11—C12—F1	119.4 (8)
C3—C4—H4	119.9	C11—C12—C13	121.0 (7)
C5—C4—H4	119.9	F1—C12—C13	119.6 (8)
C6—C5—C4	118.8 (6)	C12—C13—C14	120.0 (7)
C6—C5—H5	120.6	C12—C13—H13	120.0
C4—C5—H5	120.6	C14—C13—H13	120.0
C5—C6—C7	122.5 (6)	C13—C14—C9	119.0 (7)
C5—C6—Br1	118.6 (4)	C13—C14—H14	120.5
C7—C6—Br1	118.9 (5)	C9—C14—H14	120.5
			
C9—N1—C1—O1	3.0 (10)	C1—N1—C9—C10	138.7 (6)
C9—N1—C1—C2	−173.6 (5)	C1—N1—C9—C14	−42.7 (9)
O1—C1—C2—C3	8.0 (9)	C14—C9—C10—C11	0.3 (9)
N1—C1—C2—C3	−175.4 (5)	N1—C9—C10—C11	179.0 (6)
C1—C2—C3—C8	83.5 (7)	C9—C10—C11—F2	−177.5 (6)
C1—C2—C3—C4	−97.5 (7)	C9—C10—C11—C12	−0.6 (10)
C8—C3—C4—C5	0.9 (9)	F2—C11—C12—F1	−3.9 (11)
C2—C3—C4—C5	−178.1 (5)	C10—C11—C12—F1	179.1 (6)
C3—C4—C5—C6	0.1 (9)	F2—C11—C12—C13	177.3 (7)
C4—C5—C6—C7	−1.1 (9)	C10—C11—C12—C13	0.3 (11)
C4—C5—C6—Br1	179.1 (5)	C11—C12—C13—C14	0.1 (11)
C5—C6—C7—C8	1.1 (9)	F1—C12—C13—C14	−178.6 (7)
Br1—C6—C7—C8	−179.1 (5)	C12—C13—C14—C9	−0.4 (11)
C4—C3—C8—C7	−1.0 (9)	C10—C9—C14—C13	0.1 (10)
C2—C3—C8—C7	178.0 (6)	N1—C9—C14—C13	−178.5 (6)
C6—C7—C8—C3	0.0 (9)		

### Hydrogen-bond geometry (Å, º)

**Table:**

*D*—H···*A*	*D*—H	H···*A*	*D*···*A*	*D*—H···*A*
N1—H1···O1^i^	0.88	1.97	2.851 (7)	175
C7—H7···O1^ii^	0.95	2.63	3.309 (8)	129
C13—H13···F1^iii^	0.95	2.50	3.426 (9)	164

Symmetry codes: (i) *x*+1, *y*, *z*; (ii) *x*, *y*+1, *z*; (iii) −*x*+1, *y*+1/2, −*z*+3/2.
